# Interaction of Semaglutide and Ziprasidone in a Patient With Schizophrenia: A Case Report

**DOI:** 10.7759/cureus.59319

**Published:** 2024-04-29

**Authors:** Dustin Hejdak, Abrahim N Razzak, Lorelle Sun, Mahmudur Rahman, Pinky Jha

**Affiliations:** 1 Psychiatry, Medical College of Wisconsin, Milwaukee, USA; 2 School of Medicine, Medical College of Wisconsin, Milwaukee, USA; 3 Internal Medicine, Medical College of Wisconsin, Milwaukee, USA

**Keywords:** side effects, weight-loss drugs, schizophrenia, geodon, ozempic

## Abstract

Semaglutide (Ozempic), a GLP-1 receptor agonist effective in weight management, and ziprasidone (Geodon), an antipsychotic with a lower risk of metabolic side effects, are well-established in treating type 2 diabetes and schizophrenia, respectively. However, their interactions and effects on psychiatric symptoms are less understood. In this study, we report a case of a 43-year-old male with schizophrenia and diabetes with exacerbated paranoid delusions upon semaglutide administration for weight loss; symptoms peaked at higher doses and subsided after dose reduction. Concurrently, serum ziprasidone levels were significantly elevated at the dose reduction, suggesting a pharmacokinetic interaction likely due to semaglutide-induced slowed gastric emptying affecting ziprasidone's absorption and metabolism. This study illustrates the need for careful monitoring of psychiatric symptoms and drug levels when these medications are used together. Additionally, further research into their interactions to optimize treatment for patients with coexisting metabolic and psychiatric conditions is warranted.

## Introduction

Semaglutide, commonly known as Ozempic, has gained widespread popularity due to its efficacy in managing weight and controlling type 2 diabetes. As a glucagon-like peptide 1 (GLP-1) receptor agonist, it reduces appetite and caloric intake by mimicking incretin hormones, significantly aiding in weight management in chronic conditions including psychiatric disorders [1]. Ziprasidone, marketed as Geodon, is an atypical antipsychotic that moderates both dopamine D2 and serotonin 5-HT2A receptors with additional action as a 5-HT1A receptor agonist, characteristics that mitigate both the positive and negative symptoms of schizophrenia while presenting a low risk of metabolic side effects [2,3]. In this study, we discussed an important unusual clinical scenario that manifested due to these two medications.

## Case presentation

A 43-year-old male with a history of schizophrenia and diabetes presented to the psychotherapy clinic for medication management and weekly psychodynamic psychotherapy. The goals of therapy included decreasing irritability with his wife and his three-year-old triplet boys, as well as keeping paranoid delusions at bay. The patient was initially diagnosed with schizophrenia in his early 20s. He spent a significant amount of time in the county hospital system, trialing several different antipsychotic medications without significant improvement in his paranoid delusions and disorganized behavior. Approximately 20 years ago, patient’s symptoms finally improved after a trial of Geodon that was titrated to 80 mg nightly with meals. Of note, he developed akathisia with Geodon which has responded well to benztropine 2 mg daily, which he continues to take. He had stability for approximately 18 years on this regimen, without any medication adjustments. However, in July 2022, his primary care provider started him on Ozempic 1 mg/weekly for weight loss. Two to three weeks after the initiation of Ozempic, he began to have paranoid delusions. The paranoia worsened to the extent that the patient was unable to return to work and could only work from home. The patient denied any other changes to biopsychosocial factors that would have contributed to his psychosis. He was adamant no other variables changed in his life outside of starting the Ozempic. Given there was no literature on interactions between Geodon and Ozempic at the time, his primary care provider did not feel Ozempic was the cause and continued this medication at the prescribed dose. As such, Geodon was increased to 160 mg nightly with meals to target his psychosis. Delusions resolved approximately one to two weeks thereafter. The patient maintained stability until about seven months later, when his Ozempic dose was increased to 2 mg weekly to promote further weight loss. Approximately two weeks later, the patient had the same return of paranoid delusions. Given severity of delusions, Ozempic was decreased back to 1 mg/weekly by the primary care provider and his symptoms resolved shortly thereafter.

Given this unusual interaction, care coordination was conducted with his primary care provider, and his Geodon 12-hour serum trough level was drawn in November 2023. His level, in which he was still on the concurrent weekly 1 mg Ozempic dosage, returned to 238.7 ng/mL. The upper limit of normal was defined as 220 ng/mL. Unfortunately, a separate comparative level could not be drawn as the patient did not opt to taper off Ozempic since it has been helping with losing weight. The patient continues to do well at this time without side effects from Ozempic 1 mg weekly and Geodon 160 mg dosages. A timeline of his prescription history is detailed below (Table 1).

**Table 1:** Timeline of patient's prescription history.

Date	Prescription
2004~	Trial of various medications
2004~2005	Geodon 80 mg nightly
July 2022	Geodon 80 mg nightly and Ozempic 1 mg weekly
August 2022	Geodon 160 mg nightly and Ozempic 1 mg weekly
February 2023	Geodon 160 mg nightly and Ozempic 2 mg weekly
March 2023	Geodon 160 mg nightly and Ozempic 1 mg weekly

## Discussion

There has been some research on the effect of GLP-1 agonists and antipsychotic-treated patients with schizophrenia, one comparative study demonstrated that GLP-1 agonists promote weight control in diabetic patients on antipsychotic medications with or without antidepressants [4]. A meta-analysis showed GLP-1 agonists being effective for antipsychotic (clozapine/olanzapine) associated body weight gain [5]. Additionally, a multicenter trial is underway to investigate whether GLP-1 agonists cause adverse side effects in patients with schizophrenia [6]. However, at this time there is limited literature suggestive of interactions between Geodon and Ozempic or if Ozempic can lead to or worsen psychosis symptoms. As of now, Ozempic has been reported to have adverse effect associations for increased risk in acute pancreatitis/gallbladder disease, anesthesia complications, and potentially suicidal thoughts/behaviors [7,8]. However, long-term risks are still unknown.

Given that Geodon requires at least a 500-calorie meal to be properly absorbed, Ozempic is likely causing a type of reduced Geodon absorption [9]. One of the mechanisms for Ozempic is to slow gastric emptying which may be a potential cause of this symptomatic interaction. In this case scenario, the Geodon level in fact did return supratherapeutic at 238.7 ng/mL from the recommended maximum of 220 ng/mL. As a causality assessment, while we are unable to establish causation from this one example alone, there were no other medical histories or the presence of a likely cause for this patient's phenomenon. It is also important to note that Ozempic itself having a direct pathophysiological psychosis side effect cannot be taken off the mode of possibilities. Repeating the blood level for Geodon when patients can opt off the Ozempic to temporarily assess this interaction may be recommended for other similar clinical scenarios.

## Conclusions

This is to our knowledge the first known instance of a possible drug interaction between Ozempic and Geodon. As Ozempic continues to maintain widespread usage against the obesity epidemic, it is of utmost significance to recognize the pathophysiological interaction and presence of psychosis side effects between these two medications.
